# Super-sensitive time-resolved fluoroimmunoassay for thyroid-stimulating hormone utilizing europium(III) nanoparticle labels achieved by protein corona stabilization, short binding time, and serum preprocessing

**DOI:** 10.1007/s00216-017-0284-z

**Published:** 2017-03-16

**Authors:** Tuomas Näreoja, Jessica M. Rosenholm, Urpo Lamminmäki, Pekka E. Hänninen

**Affiliations:** 1 Laboratory of Biophysics, Institute of Biomedicine and Medicity research laboratories, University of Turku, Tykistökatu 6A, 20520 Turku, Finland; 2 Division of Pathology, Department of Laboratory Medicine, Karolinska Institutet, F46, Karolinska Universitetssjukhuset, Huddinge, 141 86 Stockholm, Sweden; 3 Pharmaceutical Sciences Laboratory, Faculty of science and engineering, Åbo akademi University, Tykistökatu 6A, 20520 Turku, Finland; 4 Department of Biochemistry/Biotechnology, University of Turku, Vatselankatu 2, 20500 Turku, Finland

**Keywords:** Sandwich-type immunoassay, Time-resolved fluoroimmunoassay, Nanoparticle bioconjugate, Immunoassay interference, Nanoparticle protein corona

## Abstract

Thyrotropin or thyroid-stimulating hormone (TSH) is used as a marker for thyroid function. More precise and more sensitive immunoassays are needed to facilitate continuous monitoring of thyroid dysfunctions and to assess the efficacy of the selected therapy and dosage of medication. Moreover, most thyroid diseases are autoimmune diseases making TSH assays very prone to immunoassay interferences due to autoantibodies in the sample matrix. We have developed a super-sensitive TSH immunoassay utilizing nanoparticle labels with a detection limit of 60 nU L^−1^ in preprocessed serum samples by reducing nonspecific binding. The developed preprocessing step by affinity purification removed interfering compounds and improved the recovery of spiked TSH from serum. The sensitivity enhancement was achieved by stabilization of the protein corona of the nanoparticle bioconjugates and a spot-coated configuration of the active solid-phase that reduced sedimentation of the nanoparticle bioconjugates and their contact time with antibody-coated solid phase, thus making use of the higher association rate of specific binding due to high avidity nanoparticle bioconjugates.

**Electronic supplementary material:**

The online version of this article (doi:10.1007/s00216-017-0284-z) contains supplementary material, which is available to authorized users.

**Graphical Abstract:**
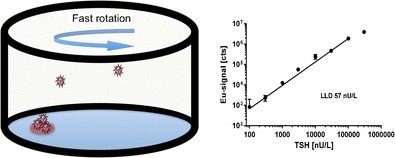
We were able to decrease the lowest limit of detection and increase sensitivity of TSH immunoassay using Eu(III)-nanoparticles. The improvement was achieved by decreasing binding time of nanoparticle bioconjugates by small capture area and fast circular rotation. Also, we applied a step to stabilize protein corona of the nanoparticles and a serum-preprocessing step with a structurally related antibody.

## Introduction

Eu-doped nanoparticles can be manufactured to afford high colloidal stability; good protection of Eu-chelates from solvent; very low self-quenching owing to long Stoke’s shift of the Eu-chelates; density that is close to water’s density to reduce sedimentation; and a dense coating of carboxyl groups on their surface for efficient bioconjugation [1]. Most importantly the Eu-doped nanoparticles facilitate sensitive time-resolved low background detection of Eu-emission and are thus perfect label candidates for assays that require high sensitivity. However, while the nanoparticle-based assay concepts have high signal output, their applicability has been limited because of assay matrix-related interference and relatively high level of nonspecific binding observed in the assays [1–3]. Matrix effects in an immunoassay usually refer to interference arising from the sample matrix (e.g., whole blood, serum, or saliva) that contains the analyte. Previously, we have used a systematic approach to characterize different aspects of a sandwich-type non-competitive immunoassay utilizing nanoparticle bioconjugates as labels [4]. We have developed a rational basis for development of immunoassays using nanoparticles as labels [5–7] and now expand this work to describe assay matrix-derived interactions [8].

Matrix-related interferences like high nonspecific binding and cross-reactivity of antibodies, autoantibodies (HAB), human anti-mouse antibodies (HAMA), and polyanions (heparin) often hamper precise measurements in clinical samples, e.g., in serum [9, 10]. In most assays, serum samples are preprocessed from whole blood by removing the blood cells and clotting factors, but they still contain a variety of factors that prevent reaching the lowest limit of detection (LLD) that can be obtained with artificial buffer samples. Binding of a nanoparticle bioconjugate is defined by nanoparticle material and the bioconjugate layer, but also by their biomolecule corona [11]. The biomolecule corona of a nanoparticle describes a loosely bound dynamic layer of non-covalently associated proteins and other biomolecules that in part mediate nanoparticle interactions [12]. TSH and thyroid hormone assays are especially prone to HAB interference, as both Graves’ and Hashimoto’s diseases are autoimmune disorders, implying the prevalence of autoantibodies to the hormones or their respective receptors. Thus, optimization of TSH assay conditions is of particular interest. There are a number of sample preprocessing strategies for various immunoassays, but none that would directly focus on removing nanoparticle corona-forming proteins.

We used TSH as a model analyte to demonstrate advances in immunoassay technology. TSH is a 28-kDa glycoprotein hormone secreted by the pituitary gland. TSH comprises two chemically different subunits, α and β, which are joined by non-covalent bonds. The normal range of TSH in serum is 0.3–5.0 mU L^−1^ (0.05–0.8 μg L^−1^), but over 95% of screened normal euthyroid volunteers have TSH levels below 2.5 mU L^−1^ [13]. Abnormal TSH level can be a sign of thyroid malfunction, and it can be used as a tool to diagnose thyroid diseases and to monitor the effectiveness of therapy. Patients suffering from hypothyroidism have elevated TSH level, and more precise TSH assays allow one to distinguish subclasses of hyperthyroidisms. Over 13 million Americans are believed to be affected by some type of thyroid disease, but thyroid diseases nevertheless remain underdiagnosed because of ineffective screening programs [14]. Furthermore, precise analysis of thyroid function is required during pregnancy, as one to three in 200 pregnancies are affected by thyroid dysfunction caused by the autoimmune disorders Graves’ and Hashimoto’s diseases [15, 16]. These conditions are dangerous and may cause congestive heart failure for the mother, miscarriage, and attention deficit hyperactivity disorder symptoms or impaired cognitive development for the child. The likelihood of adverse effects is increased especially if the thyroid disease develops during the first trimester [16, 17]. Furthermore, there is increasing evidence on thyroid dysfunction being one of the factors triggering or aggravating metabolic syndrome [18, 19]. Slightly elevated TSH levels have been linked to metabolic syndrome, also in euthyroidism; in particular, young females with a TSH in the upper normal range (2.5–4.5 mU L^−1^) were more likely to be obese, had higher triglyceride levels, and were more likely to be affected by metabolic syndrome.

Good precision and accuracy are often problematic to achieve at the same time, and therefore small changes in trends cannot usually be detected in a number of diagnostic test formats. Hence, more powerful tools are required to quantitatively detect low concentrations of target analyte, small changes in concentrations, and trend reversals due to intervention therapy [19]. In plasma and serum the matrix-related interferences often hamper precise measurements, especially in the case of autoimmune disorders [20]. In this study we demonstrate the effects of our conceptual findings on the TSH immunoassay performance, and especially their potential to reduce matrix interference. Our optimized europium(III) nanoparticle labeling technology in sandwich-type immunoassays is shown to improve the LLD to 60 nU L^−1^, which equals 450 amol L^−1^ [21], and is 1/50 of the LLD obtainable by the current market leaders in TSH assays. The assay is based on 96 well-plate format, where Eu(III)-labeled nanoparticles are used as labels and time-resolved fluorescence is used to monitor TSH quantitatively from the surface of a reaction well.

## Experimental

### Reagents

Europium(III)-chelate-doped Fluoro-Max™, carboxyl-modified, monodisperse, polystyrene nanoparticles with a 92-nm diameter were acquired from Seradyn (Indianapolis, IN). The fluorescent properties of these particles were described previously [22, 23]. The particles are stabile in aqueous suspensions and nanoparticle material protects doped Eu-chelates, thereby facilitating stabile fluorescence [24]. Anti-TSH monoclonal antibodies (clones anti-TSH 5404 SP-1 and anti-TSH 5409 SPTNE-5) were purchased from Medix Biochemica (Kauniainen, Finland). Antibody fragments (anti-prostate specific antigen (PSA) Fab 5A10, Fab anti-TSH 5409, and anti-TSH 5404) were produced at the Department of Biotechnology in the University of Turku [25, 26]. Affinity constants for the antibodies were 9.3 × 10^8^ L mol^−1^ and 2.2 × 10^10^ L mol^−1^ for anti-TSH 5409 and anti-TSH 5404, respectively [27]. KaivogenSA96™ streptavidin-coated microtitration low-fluor plates, KVG buffer [50 mmol L^−1^ Tris–HCl (pH 7.8), 150 mmol L^−1^ NaCl, 7.7 mmol L^−1^ NaN_3_, 76 μmol L^−1^ bovine serum albumin, 80 μmol L^−1^ Tween 40, 3 μmol L^−1^ bovine γ-globulin, 20 μmol L^−1^ diethylenetriaminopentaacetic acid] and washing solution (5 mmol L^−1^ Tris–HCl, pH 7.8 containing 150 mmol L^−1^ NaCl, 3.5 mmol L^−1^ Germall II, and 40 μmol L^−1^ Tween 20) were from Kaivogen Oy (Turku, Finland). *N*-Hydroxysulfosuccinimide (NHS) was acquired from Fluka (Buchs, Switzerland); bovine serum albumin fraction V (BSA), biotin, and *N*-(3-dimethylaminopropyl)-*N*′-ethylcarbodiimide (EDC) were purchased from Sigma (Steinheim, Germany).

### Conjugations and coatings

**Fig. 1:**
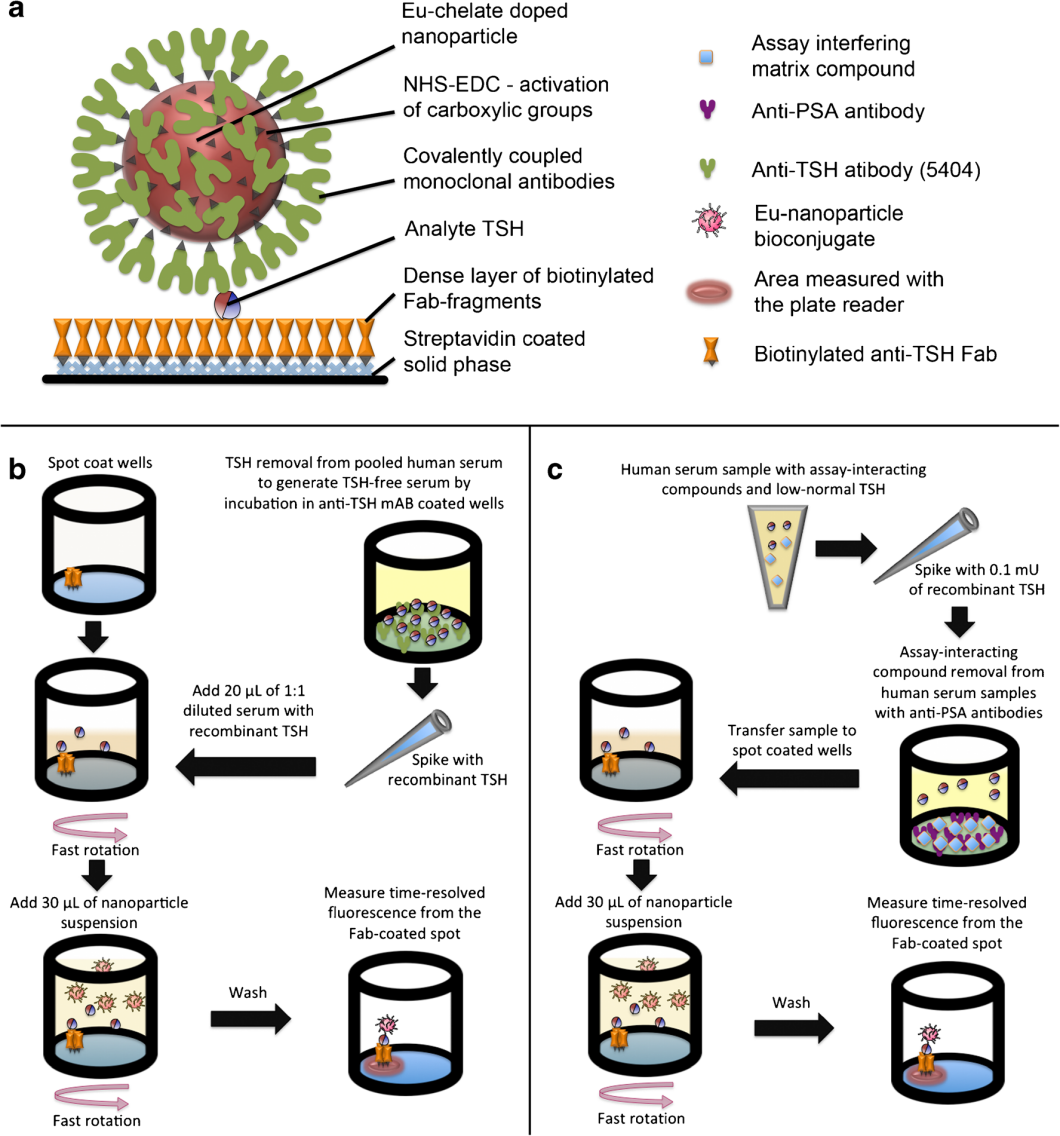
Schema and flow diagram of the assay concept. **a** Sandwich complex. Carboxyl groups on Eu-doped polystyrene nanoparticles are functionalized with EDC-NHS (treatment to generate succinimidyl groups). Then the particles are coated with monoclonal anti-TSH antibodies to form the nanoparticle bioconjugates and unreacted succinimidyl groups are converted back to carboxylic acid through hydrolysis. The solid phase is first coated with streptavidin and blocked with BSA; subsequently, an area of approximately 1 mm^2^ is functionalized with anti-TSH Fab fragments recognizing a different epitope in TSH than the nanoparticle-conjugated antibodies. **b** To optimize the assay we needed TSH-free human serum and we produced it by affinity purification of pooled human serum. For the purification we used 2 × 10 min incubation of the serum in anti-TSH-coated microtiter wells, after which we spiked samples with known concentrations of recombinant human TSH. The TSH was added to serum into equal volume of KVG buffer, and 20 μL of this mixture was added to spot-coated wells. After 10 min incubation with fast circular mixing the nanoparticle bioconjugates were added in a volume of 30 μL and incubation was continued for 30 min. Subsequently, unbound nanoparticle bioconjugates were washed away, and wells were read for time-resolved Eu-fluorescence. **c** To reduce nonspecific binding in a nanoparticle-based immunoassay we used a nonspecific affinity purification step to remove compounds interfering with the assay. First, a sample of female serum low–normal range TSH sample was spiked with 100 μU L^−1^ of TSH in KVG buffer (Fig. 4) and incubated for 2 × 10 min in anti-PSA Fab-coated wells (women do not have PSA in circulation so nothing specific is removed). A replicate sample was spiked, but left untreated. 20 μL of both samples were transferred to spot-coated wells and assayed like in protocol **b**. Difference in TSH-assay performance is presented in Fig. 4

Nanoparticles were covalently coated with Mab anti-TSH 5404 according to a previously described procedure using 6 μmol L^−1^ Mab [22] (Fig. 1). The monoclonal antibodies were biotinylated randomly through lysines according to a protocol described earlier [26]. The Fab fragments were produced, site-specifically biotinylated, and coated on streptavidin-functionalized solid phase according to a previously described procedure [5]. The nanoparticle bioconjugates were diluted 5- to 1000-fold into KVG buffer or a subset of the buffer’s components and incubated for at least 24 h to allow their protein corona to re-equilibrate [8].

The spot-wells were produced by washing streptavidin-coated microtitration wells twice with the washing solution to remove preservatives and loosely bound proteins. To produce fully coated wells 3 × 10^−13^ mol of biotinylated Fab fragments were incubated in the prewashed wells in 30 μl of KVG buffer for 20 min. In spot-coating 1 × 10^−13^ mol of biotinylated Fab fragment was incubated in 1 μL of KVG buffer for 10 min. The spot was produced by pipetting a drop of capture antibody solution halfway between the edge and center of the well of a streptavidin-functionalized 96-well plate. The majority of the solid phase is thus left devoid of the capture antibody, and binding of the nanoparticle bioconjugate on it will thus be significantly lower (Fig. 1b) [8].

### Immunoassays

Known concentration of TSH was spiked in KVG buffer and incubated for 20 min. Alternatively, the TSH was mixed into affinity purified serum/KVG buffer mixture and incubated for 10–20 min (Fig. 1). In the one-wash assay configuration TSH was spiked into a 1:1 mixture of affinity-purified pooled serum and KVG buffer, and 20 μL of this mixture was then transferred to a reaction well. After 10 min of incubation under fast circular shaking (DELFIA Plateshake 1296-003, with a circular vibrating motion, PerkinElmer), 3 × 10^7^ detector nanoparticle bioconjugates in 30 μL of KVG buffer were added to the reaction wells and incubated for another 30 min under fast circular shaking. Subsequently, the wells were washed six times and aspirated. The time-resolved fluorescence from the nanoparticle–antibody bioconjugates was measured by excitation at 340 nm and detection at 615 nm using time-resolved fluorescence of plate reader Victor^2^ 1420 Multilabel counter (Wallac, PerkinElmer) [8].

### Affinity purification

Serum was isolated from peripheral blood of healthy volunteers (*N* = 7; 3 male, 4 female) who gave their informed written consent. Affinity purification was performed by incubating pooled human serum in microtiter wells coated with 50 μL of 100 nmol L^−1^ anti-TSH Mab 5404 (Fig. 1b). Alternatively, the serum was purified with an antibody against prostate specific antigen (PSA) anti-PSA Fab 5A10 (50 μL of 200 nmol L^−1^) that was, apart from the paratope, structurally similar to the anti-TSH antibodies (Fig. 1c). In this experiment a female donor’s serum was used because of lack of PSA in the circulation. The donors’ (*N* = 2) TSH was tested to be below 1 mU L^−1^ 2–4 weeks before the serum sample for this study was taken. The incubation lasted 15 min, and the serum was treated twice in a volume of 60 μL. The purified serum was extracted from wells and stored refrigerated or frozen until used. We used regular two-way ANOVA with Sidak’s test for multiple comparisons available in GaphPad Prism to test significance of the observed difference between affinity-purified samples and untreated samples.

### Dynamic light scattering measurements

The nanoparticle bioconjugates were vortexed, diluted into KVG buffer, sonicated, vortexed again, after which the dynamic light scattering (DLS) was measured immediately (Zetasizer Nano, Malvern Instruments, Worcestershire, UK). The stabilized samples were diluted, sonicated, vortexed, and allowed to re-equilibrate their protein corona for 24 h, after which the samples were sonicated and vortexed again and DLS was measured.

## Results

### Stabilization of protein corona on nanoparticle bioconjugates

**Fig. 2:**
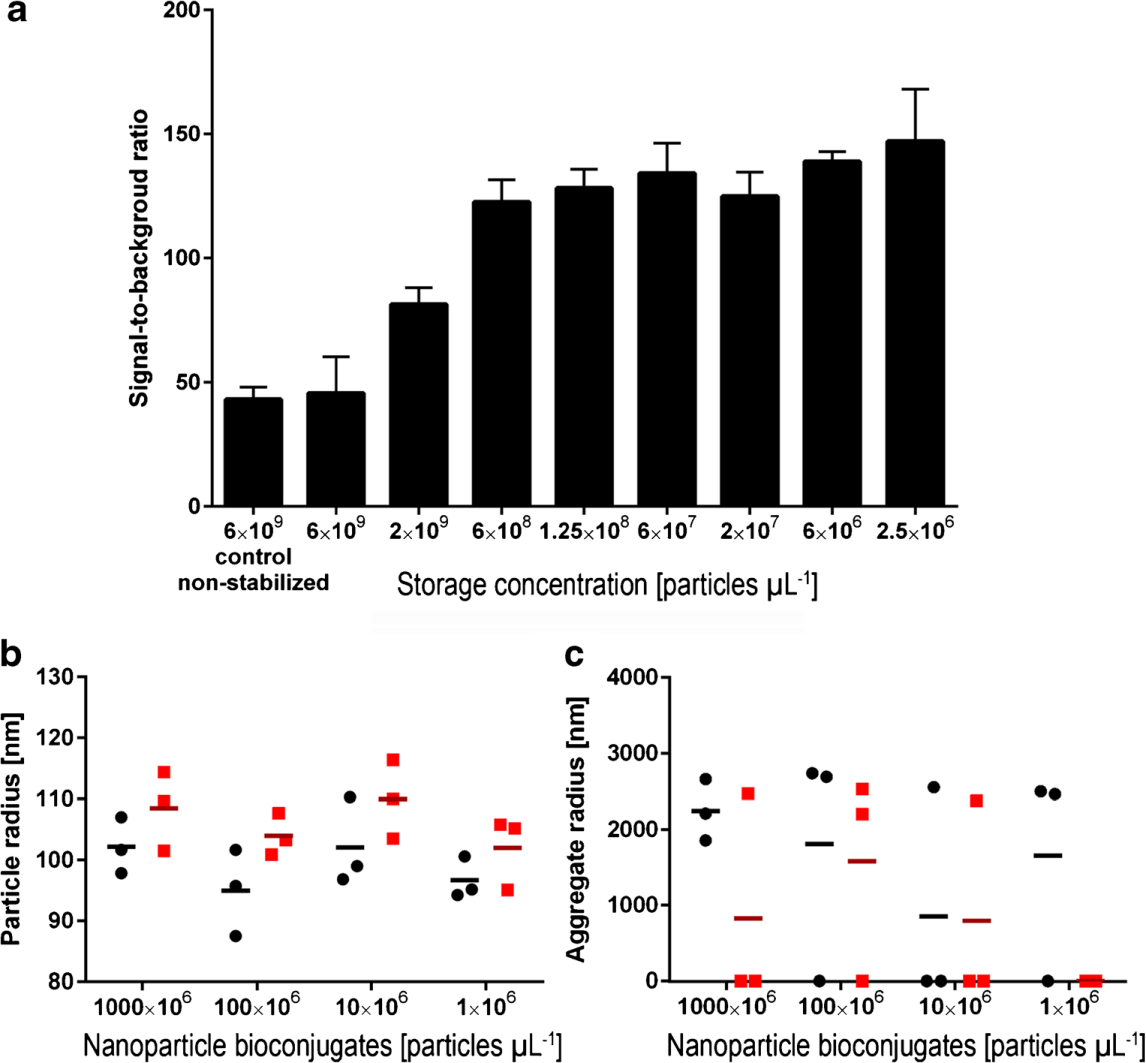
Concentration-dependent protein corona stabilization. **a** The nanoparticle bioconjugates were stored in KVG buffer at various concentrations and then used to perform a one-step assay for 100 μU L^−1^ TSH in KVG buffer assay matrix. Dilution to the assay concentration (2.5 × 10^6^ particles μL^−1^) was made in a single step. Stabilization of the particle suspension was achieved up to a concentration 100-fold higher than the concentration used in the assay. Mean values of three replicates are presented as *bars* and standard deviation as *error bars*. **b** Main peak of a DLS measurement of nanoparticle bioconjugates diluted into KVG buffer measured immediately after dilution, sonication, and vortexing (*black circles*, *black line* represents a mean of 3 replicates) and one measured after dilution, 24 h stabilization, sonication, and vortexing (*red squares*, *deep red line* represents a mean of 3 replicates). **c** Representation of the aggregate peak of the same DLS measurement; while we observed aggregates only in some of the samples, typically the aggregates were fewer and smaller in the stabilized samples. A zero value indicates that the amount of aggregates in the sample fell below the detection limit

The colloidal stability of nanoparticle bioconjugates in suspension is determined by their surface potential [28], stability of conjugated antibodies [29], ionic strength and pH of the assay buffer [30], concentration of nanoparticle bioconjugates in the suspension, detergents in the assay buffer, presence of blocking proteins, and stability of their protein corona [12]. In contrast to small molecular labels attached to antibodies that have a miniscule influence on their microenvironment, the nanoparticles form a polarized solvation shell near the surface of the particle where exchange of molecules will be slower [8]. As the nanoparticle bioconjugates are typically diluted in the assay buffer from a separate storage buffer, i.e., stock suspension, the equilibrium of proteins loosely adhered to their surface is perturbed. During formation of the new equilibrium and molecule exchange on the nanoparticle surface, low energy bonds (e.g., electrostatic and van der Waals) form and break, and this may generate unwanted nonspecific interactions [31–33]. We discovered that storing nanoparticle bioconjugates in KVG buffer (a buffer with blocking proteins and detergent) at a concentration of no higher than 100 times that of the final usage concentration in the following assay increased the signal-to-noise ratio of the assay by nearly threefold (Fig. 2a). This can be explained by agglomeration of nanoparticle bioconjugates upon coming into contact with the high protein concentration in the serum matrix [34] or the assay buffer, as they are diluted from a high storage concentration. We examined the process by dynamic light scattering (DLS) experiments where nanoparticle bioconjugates were diluted in KVG buffer and measured immediately or after 24 h protein corona stabilization. We observed an increase of hydrodynamic radius of single particles corresponding to the adsorption of matrix proteins onto the nanoparticle bioconjugates and formation of a new protein corona (Fig. 2b). Furthermore, we observed both fewer and smaller aggregates in the stabilized samples (Fig. 2c). The aggregates represented a particle volume below 5% of the total DLS signal; hence, these aggregates were difficult to measure, resulting in high variation of signal between the replicate samples. The controlled reformation of the protein corona (Fig. 2b) appears to reduce agglomeration-inducing interactions between particles (Fig. 2c), and as a result the immunoassay had higher specific signal, as more binders were available, and lower nonspecific signal (Fig. 2a), as prevalence of large aggregates prone to sediment is reduced.

**Fig. 3:**
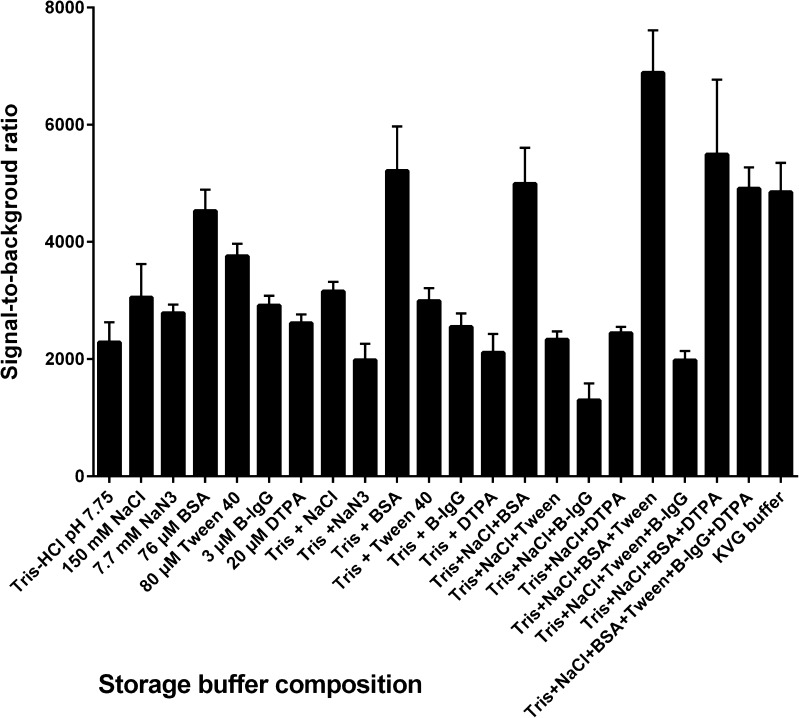
Component-by-component investigation of the storage buffer composition at concentration of 1.25 × 10^8^ particles μL^−1^ and 1 mU L^−1^ of TSH. Mean values of three replicates are presented as *bars* and standard deviation as *error bars*. Impact of the storage buffer composition is measured by testing the assay performance (signal-to-background ratio) with the particles stored in the specified buffer and then diluted into KVG buffer. The best signal-to-background ratio was obtained with particles stored in 50 mmol L^−1^ Tris–HCl (pH 7.8), 150 mmol L^−1^ NaCl, 76 μmol L^−1^ bovine serum albumin, and 80 μmol L^−1^ Tween 40

Components in the protein corona may improve colloidal stability and increase the activity of the nanoparticle bioconjugates by reducing unwanted interactions or decrease their binding activity by masking binding sites [32]. To investigate the stabilization effect in more detail we did a component-by-component test of the KVG buffer ingredients to discover the components responsible for the effect. Also, we wanted to reveal whether the effect was due to more dispersed particle suspension after the dilution rather than due to any specific buffer components. The test of the buffer ingredients was performed at a particle concentration of 1.25 × 10^8^ particles μL^−1^ (Fig. 3) [8] . The most critical storage buffer component regarding the stabilization was the blocking protein BSA, and the second most important was detergent Tween 40. The importance of the detergent was less pronounced when buffer pH and salinity, i.e., ionic strength, were adjusted to physiological range. Stabilization using the optimum pH, physiological ionic strength, blocking protein, and detergent in the diluted particle suspension resulted into a nearly threefold improvement in signal-to-background ratio. However, when these components were added to the storage buffer at a particle concentration 1.25 × 10^10^ particles μL^−1^, no benefit was observed. This would indicate that the stabilization effect is dependent on the surface kinetics, as increasing the concentration of stabilizing agents to match the surface area did not provide a similar result.

### Control of association time by solid-phase organization

**Fig. 4:**
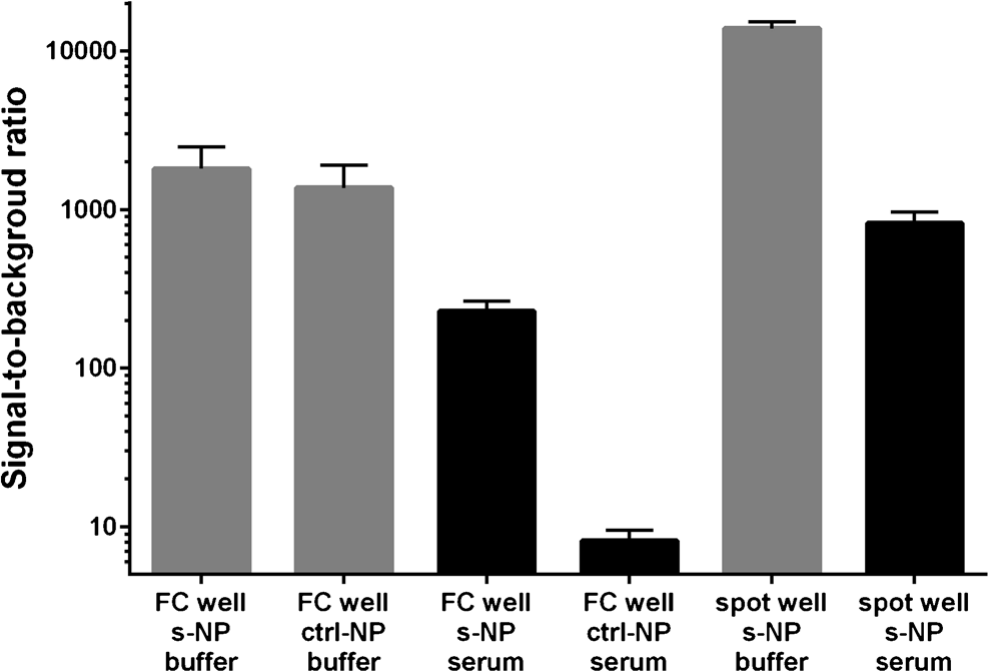
Comparison of TSH assay signal-to-background ratios in KVG buffer (buffer, *gray bars*) and in 50% affinity-purified serum (serum, *black bars*). TSH concentration was 100 μU L^−1^; mean values of three replicates are presented as *bars* and standard deviation as *error bars*. The assay utilized normal (ctrl-NP) and protein corona-stabilized nanoparticle bioconjugates (s-NP) and fully coated (FC) and spot-coated (spot) microtiter wells. The optimized configuration gave 10-fold higher signal-to-background ratio in KVG buffer configuration with fully coated well and control NPs and a 100-fold higher ratio in 50% affinity-purified serum configuration with fully coated well and control NPs

We have shown with force spectroscopy and surface plasmon resonance that the association rate of specific binding of nanoparticle bioconjugates (*k*_on_) is up to 200,000-fold higher than *k*_on_ for nonspecific binding, while *k*_off_ for both specific and nonspecific binding was too slow to be measured [7]. Also, nonspecific binding occurs mainly between two antibody-coated surfaces [5] and is dependent on the density and stability of the antibodies [6]. We were able to show that colloidal stability is critical for assay performance (Fig. 2); to further reduce sedimentation and nonspecific binding, we decided to reduce the binding time of the nanoparticle bioconjugates with fast liquid flow induced by mixing and a small area for specific binding (Figs. 1 and 4) [35, 36]. The small area of the active solid phase was produced by adding the Fab capture antibody in a 1-μL drop on a well of a streptavidin-functionalized 96-well plate, halfway between the edge and center of the well. At this location the liquid flow induced by circular shaking was the fastest and no edge effect could be expected. Thus, the majority of the solid phase was left devoid of the capture antibody. Binding of the nanoparticle bioconjugate towards the streptavidin-coated and BSA-blocked surface was 10–500 times less efficient than to antibody-coated surface [5, 23]; hence, a lower level of background signal was observed with zero TSH calibrator samples. The spot-coated wells provided 3- to 10-fold higher signal-to-background ratio than the fully coated wells (Fig. 4). Moreover, the assays in spot-coated wells required lower concentration nanoparticles to produce optimal signal level (see Electronic Supplementary Material (ESM) Fig. S1), because we directed the excitation beam of the Victor^2^-multilabel counter to the coated spot and achieved a strong excitation of the specifically bound nanoparticle bioconjugates. The nonspecific signal is directly proportional to the number of nanoparticle bioconjugates used in the assay, so this in part helped to reduce the background signal [4].

### Sample preprocessing by affinity purification

**Fig. 5:**
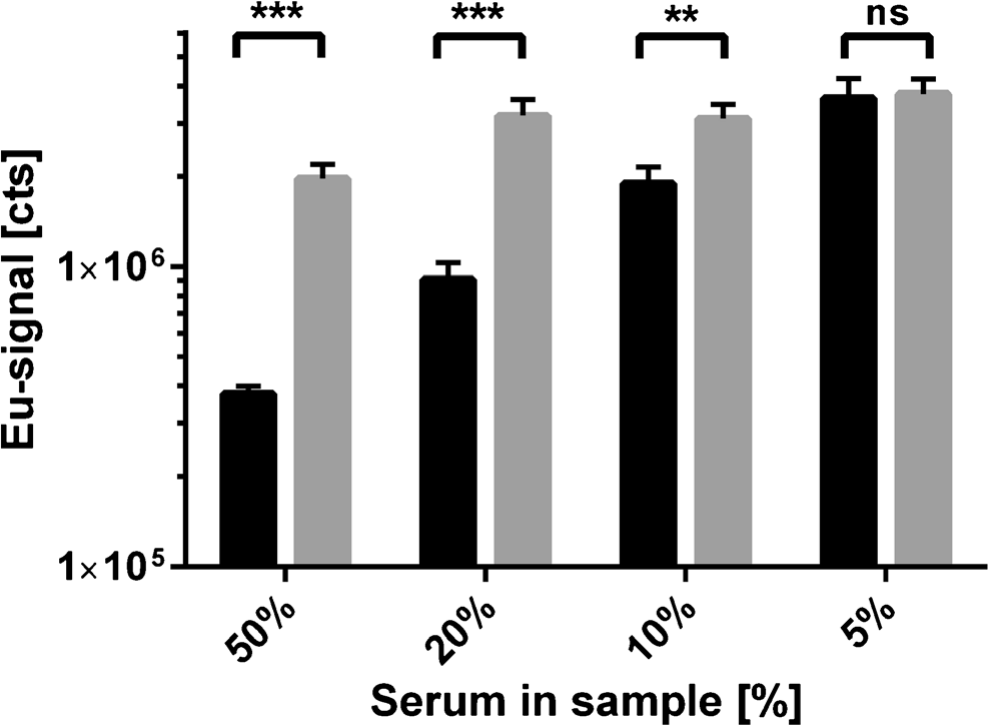
Affinity purification increases the sensitivity of the assay. We added (100 μU L^−1^) of TSH (Sample + 100 μU L^−1^) into female serum samples with low–normal range TSH (Sample) (approximately 0.4–1 mU L^−1^). TSH recovery was illustrated by plotting Eu-signal (Sample + 100 μU L^−1^) – Eu-signal (Sample). The serum samples were not purified (*black*) or affinity purified (*gray*) with Fab 5A10 anti-PSA antibodies that beared a resemblance to anti-TSH antibodies, but having no specific antigens in the serum. Addition was done in respect to total well volume, and thus all samples should have given the same Eu-signal. Differences between treated and untreated samples were tested with regular two-way ANOVA with Sidak’s test for multiple comparisons; *ns p* > 0.05, **p < 0.01, and ***p < 0.001

The normal range of TSH in patient samples is approximately 0.4–2.5 mU L^−1^. To measure a TSH calibration curve, the serum samples were prepared from pooled serum samples by affinity purification with anti-TSH antibodies, and subsequently these samples were spiked with recombinant TSH (Fig. 1b). We observed that the Eu-signal measured from zero TSH calibrator of TSH-stripped serum samples was lower than that measured from buffer-only samples (Fig. 4). Analyzing these samples we observed a reduced and reproducible level of nonspecific background signal in the zero TSH calibrator sample (Fig. 4). This implies that the protocol used to remove TSH from the pooled serum also removed other cross-reactive or interfering compounds from the sample. Removal of such compounds from a clinical sample would naturally enable detection of even lower concentrations of TSH. We then asked if it would be possible to remove these interfering compounds with a pretreatment using an antibody that does not recognize anything specific in the serum samples. To quantify this observation, we utilized anti-PSA 5A10 Fab fragment [5]-coated wells to purify pooled female serum samples with low-normal range TSH (0.4–1 mU L^−1^) after they had been spiked with (100 μU L^−1^) recombinant TSH (Fig. 1c). The anti-PSA Fab was produced in a similar manner as the anti-TSH Fab fragment, and the amino acid sequence was over 90% similar. However, in female serum samples there should not exist any epitopes that the anti-PSA Fab fragment would recognize, and therefore the observed affinity purification was not caused by removal of any specific interacting compound. After the purification step, we measured recovery of spiked recombinant TSH 100 μU L^−1^ in samples with 50%, 20%, 10%, and 5% of serum (Fig. 5). The TSH concentration of all the samples was equal, and only the amount of serum differed. Therefore, with perfect recovery of recombinant TSH, the signal in all measurements should be equal to 5% sample. The observed decrease of Eu-signal represents reduced TSH recovery due to matrix-related effects. We observed that at serum quantity over 5% (v/v) the affinity purification produced a significant increase of TSH recovery (Fig. 5). The effect of affinity purification was stronger the higher the proportional amount of serum in the sample was.

### Super-sensitive TSH assay utilizing nanoparticle bioconjugates

**Fig. 6:**
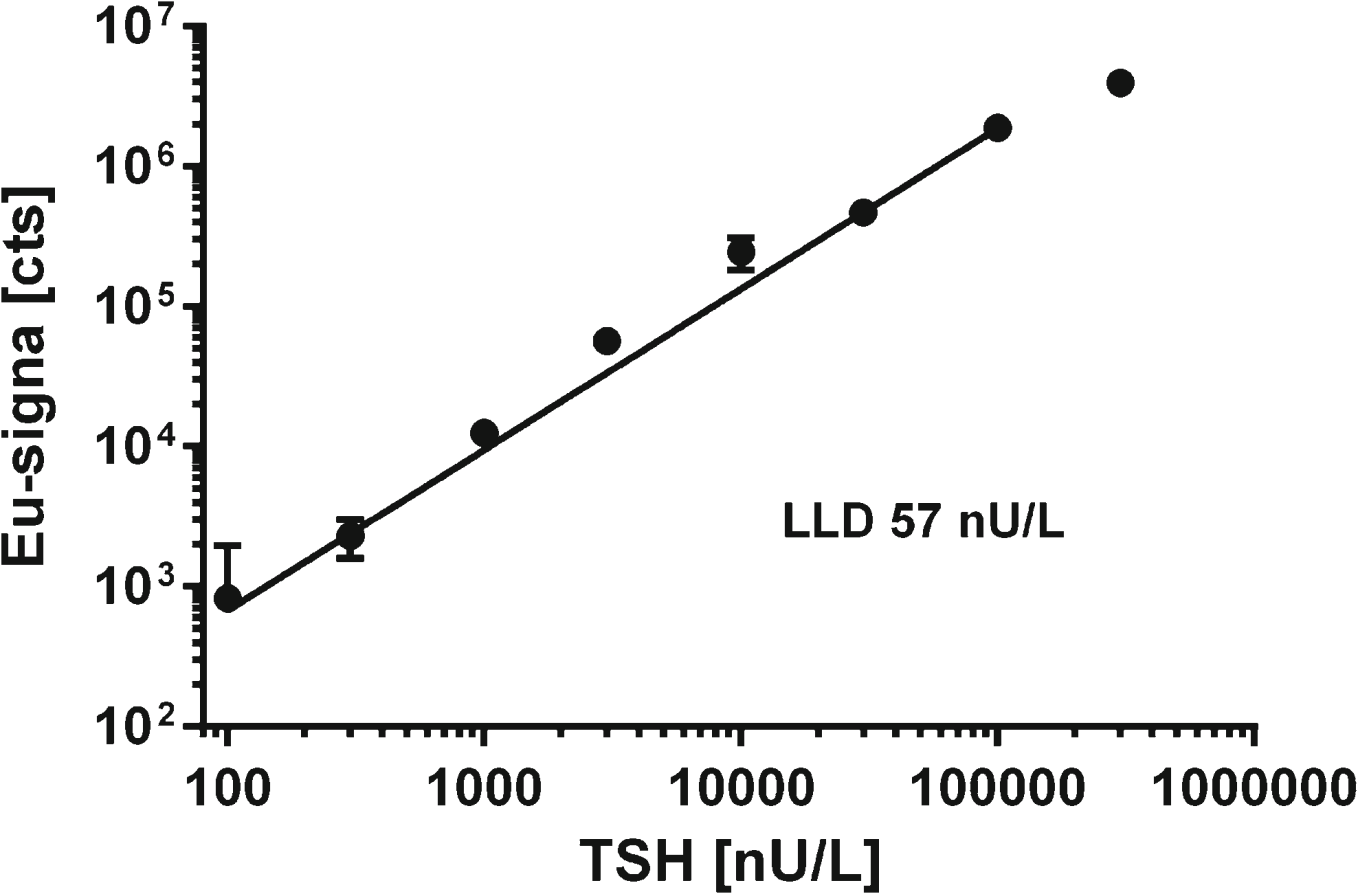
TSH standard curve measured in spiked affinity-purified serum samples by using three replicates of each calibrator. The LLD of the assay was 60 nU L^−1^ corresponding to 450 aM or 10,000 molecules in sample volume of 20 μL. Background signal was subtracted from the data points presented and the average background signal is set to zero value and LLD at 3 × SD of background

We used our findings to develop a heterogeneous sandwich-type immunoassay utilizing nanoparticle bioconjugates to measure TSH in affinity-purified pooled human serum. A standard curve measured using three replicates of each calibrator reached an LLD of 60 nU L^−1^ (Fig. 6); this corresponds to 450 amol L^−1^ or 10,000 molecules in a sample volume of 20 μL. The LLD was defined as the signal of 3 × SD of the zero calibrator over the signal obtained from zero calibrator. Furthermore, in the presented assay the slope of signal increase for each 1 μU of TSH in serum was 13,500 Eu-signal units. The high sensitivity (signal increase per unit of TSH) of the assay enabled high precision in the determination of a patient’s TSH level.

The assay is a one-wash configuration where the undiluted sample is first dispensed into a microtiter well and thereafter tracer nanoparticles are added to the same well without a separation step in between. A single washing step is required before the time-resolved luminescence signal is recorded. The presented assay is relatively easy to perform, total time to conduct the assay is 40 min, and it requires low amounts of sample and reagents, although the affinity purification increases slightly with the amount of antibodies used. We were able to operate the assay with serum volumes of 5–40 μL. Further reduction of sample volume induced a non-linear reduction in measured LLD. There was no kinetic requirement for the incubation period, but we chose to measure the signal when equilibrium was reached for the binding reaction because we wanted the principles presented to be more universally applicable.

## Discussion

Our longstanding aim has been to establish general principles for developing sandwich-type immunoassays utilizing Eu-nanoparticle labels [4–8, 23]. Here we applied those principles, focusing on the matrix-related nonspecific binding and developed a sandwich-type heterogenic immunoassay for TSH that has a lower LLD and higher sensitivity than previously presented assays. The LLD of 60 nU L^−1^ in our assay is nearly 1/50 of that of the market-leading assay systems: the chemiluminescence microparticle immunoassay (CMIA) 2.5 μU L^−1^ by Architect i2000 SR (Abbott Diagnostics) or the electrochemiluminescence immunoassay (eCLIA) 5 μU L^−1^ by Cobas 6000 (Roche Diagnostics). Furthermore, we introduce a concept of stabilization of the protein corona surrounding the nanoparticle bioconjugates that reduced nonspecific binding, especially in clinically relevant matrixes. The presented assay concept will facilitate better recovery of TSH from serum and more precise TSH measurements, and thereby smaller sample volumes. The observed increase in recovery suggests that there are components preventing interaction of nanoparticle label and TSH in the serum matrix. Furthermore, the observed decrease of background signal after the antibody capture-based removal of TSH implies that some of these interacting components cause the increase of nonspecific binding. Although we have not defined the interacting compounds, we suggest that a preprocessing step comprising an affinity purification with an antibody not binding the analyte or matrix filtration (Fig. 5) would increase the performance of many clinical immunoassays and would help to remove both false positives and negatives [20, 37, 38]. We hypothesize that removal of interfering compounds would be especially important in samples where HABs are likely to be present [15]. Furthermore, we propose that the best result of affinity purification is likely to be produced by an antibody resembling the capture antibody that has structural differences only in the hypervariable loops of the paratope.

The assay performance was further improved with of spot-coated configuration of the active solid phase and stabilized protein corona surrounding the nanoparticle bioconjugates. Dilution of the nanoparticle bioconjugates into another buffer or assay matrix causes a change in their protein corona, i.e., proteins loosely adhering to the nanoparticles [12, 33]. Rapid change in the protein corona may cause aggregation of the nanoparticles, as macromolecules in the matrix transiently interact with multiple nanoparticles. These interactions may also mask some of the binding sites, if a dense corona is electrostatically attracted to surround a nanoparticle (Figs. 2 and 3) [8]. These effects can be avoided by incremental dilution of nanoparticles and maintaining adequate concentration of stabilizing compounds (Fig. 3) and allowing time for the protein corona to stabilize before the assay in conducted (Fig. 2). Moreover, we provide evidence that the stabilization effect is independent on nanoparticle concentration and that re-formation of the protein corona can be observed with DLS (Fig. 2). The benefit of spot-coated solid-phase configuration (Figs. 4 and 6) is likely to originate from reduced rolling of nanoparticles on an antibody-coated surface [6] that would potentially increase nonspecific binding by allowing more time for the nanoparticle bioconjugates to adhere via nonspecific bonds. Also, the configuration concentrates the analyte in a confined area promoting efficient multivalent recognition by the nanoparticle. Yet another mechanism through which an improved signal-to-background ratio could be mediated is the decrease in the optimal amount of nanoparticle bioconjugates needed for the assays done in spot-coated wells (see ESM Fig. S1). Our assay concept, however, could not be applied to heparin plasma, most likely because of interactions caused by the polyanions. The ability to perform a washing-free immunoassay in a whole blood sample smaller than 20 μL can be seen as a prerequisite for patient self-testing and point-of-care systems testing for biomarkers in the circulation [39]. While we present a heterogeneous test, the LLD measured is sufficient to design a separation-free or homogenous system that typically performs at an order of magnitude higher LLD or better. We suggest that the principles found in this study are applicable to, e.g., lateral flow-based systems.

The higher the sensitivity of the assay is, the smaller the concentration differences that can be measured. Such a property is of importance when assessing drug response and appropriate dosage, especially in rapidly developing conditions like pregnancy-related thyroid dysfunctions where both too high and too low thyroid hormone concentrations pose a risk for the fetus and the mother [16, 17, 40]. These conditions require exact and repeated measurements to adjust the drug dosage over the course of pregnancy [41], and in some cases treatment needs to be started to promote fertility [40]. Furthermore, thyroid hormones modulate many metabolic pathways relevant to the resting energy expenditure, and hypothyroidism is associated with weight gain and metabolic syndrome [42]. Recognizing trend reversals is essential in the treatment of metabolic syndrome [19, 43].

## Conclusions

A super-sensitive time-resolved fluoroimmunoassay for TSH, LLD 60 nU L^−1^, utilizing europium(III) nanoparticle labels with reduced nonspecific binding was developed. An affinity purification step with an antibody bearing close resemblance to the detecting and capture antibody removed interfering compounds from the sample matrix [8]. Combining these properties with previously found general parameters in sandwich immunoassays [5–7] facilitated detection of TSH concentrations that were 1/50 of LLD of the current market-leading technologies (Fig. 6) and increased sensitivity [21]. With more accurate diagnostics, intervention therapy can start earlier, and total health care costs can be reduced. However, precision and accuracy are often problematic in a number of test formats, and small changes in trends cannot be detected. There is continuing demand for high-performance clinical tests, and central laboratories still process the majority of diagnostic tests owing to lower unit costs [39, 44].

## Electronic supplementary material

Below is the link to the electronic supplementary material.

ESM 1 (PDF 134 kb)

## Abbreviations

HAB: Human autoantibody
HAMA: Human anti-mouse antibody
LLD: Lowest limit of detection
TSH: Thyroid-stimulating hormone
