# Supplementary material for: Super-sensitive time-resolved fluoroimmunoassay for thyroid-stimulating hormone utilizing europium(III) nanoparticle labels achieved by protein corona stabilization, short binding time, and serum preprocessing
Source: Anal Bioanal Chem. 2017 Mar 16;409(13):3407–16. doi: 10.1007/s00216-017-0284-z (PMC5395595; doi:10.1007/s00216-017-0284-z)
Supplement: Supplementary file 1 — (PDF 134 kb) [file 216_2017_284_MOESM1_ESM.pdf]

Analytical and Bioanalytical Chemistry

Electronic Supplementary Material

Super-sensitive time-resolved fluoroimmunoassay for thyroid-stimulating hormone utilizing europium(III) nanoparticle labels achieved by protein corona stabilization, short binding time and serum preprocessing

Tuomas Näreoja, Jessica M. Rosenholm, Urpo Lamminmäki, Pekka E. Hänninen

Supplementary methods

The amount of nanoparticle bioconjugates in assays performed in spot-wells was optimized (Fig. S1). Since the spot-wells concentrated the binding events to the measurement area, fewer particles could be used to provide higher signal than in whole coat wells. Using fewer particles reduced the nonspecific signal even further. Nanoparticle amount 3×10^7 / well was used in the optimized spot-well immunoassay.

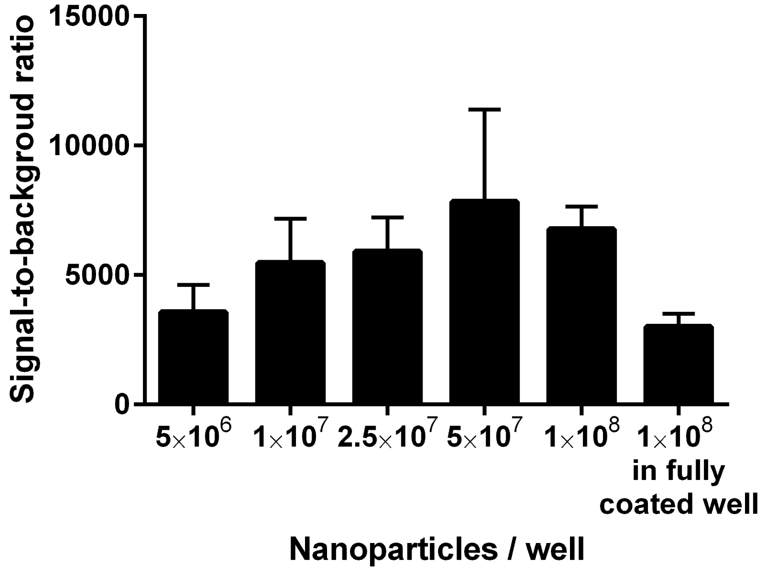

Fig. S1 Optimal amount of nanoparticle bioconjugates in the spot-coated wells determined by measuring the signal-to-background ratios. The optimal amount nanoparticle bioconjugates was 3-fold less than in the case of fully coated wells

The LLD was calculated by first subtracting average background signal from the calibrator sample averages. The standard deviation of the background replicates (N=5) was calculated. After that we fit a power-function (1) to the background subtracted signal values.

(1) $$y = ax^b$$

From parameters of this equation the LLD (2) was set to be 3×SD of background signal.

(2) $$LLD = \left(\frac{3\times SD}{a}\right)^{\frac{1}{b}}$$

LLDs in for four independent standard curves were in the range 57 – 150 nU L^{-1}, of these standard curves we show the best one in the manuscript.
